# No evidence for competition over floral resources between winter-active parasitoids and pollinators in agroecosystems

**DOI:** 10.1038/s41598-024-52146-9

**Published:** 2024-01-26

**Authors:** Lucy Alford, Sacha Roudine, Dimitra Valsami, Tiphanie Fontaine-Guenel, Talay Namintraporn, Anaëlle Guedon, Romane Normand, Ludovic Lagneau, Cecile Le Lann, Joan Van Baaren

**Affiliations:** 1 School of Biological Sciences, University of Bristol, 24 Tyndall Avenue, Bristol, BS8 1TQ UK; 2 University of Rennes, CNRS, ECOBIO [(Ecosystems-Biodiversity-Evolution)]-UMR 6553, Campus de Beaulieu, 263 Avenue du Général Leclerc, 35042 Rennes Cedex, France

**Keywords:** Agroecology, Ecosystem services

## Abstract

Warming temperate winters are resulting in increased insect winter activity. With modern agroecosystems largely homogenous, characterised by low floral diversity, competitive interactions may arise between flower-visiting species, with potential implications for the ecosystem services they provide (e.g. biological control and pollination). Flower strips may be implemented during winter months to support flower-visiting insects and enhance ecosystem service provision. Employing field trials conducted in Brittany, France between 2019 and 2021 and laboratory cage experiments, the current study examined the impact of winter flower strips on aphid biological control performed by parasitoid wasps and the potential for competitive interactions between winter-active parasitoids and pollinators. Results revealed that parasitism rate was not enhanced by the presence of winter flower strips. This lack of effect was not the consequence of pollinator presence, and the current study found no effect of pollinator abundance on parasitism rate. Flower strips may thus be implemented during winter months to support nectar-feeding insects when floral resources are scarce, with no evidence of exploitative competition between pollinators and parasitoids, nor a detrimental impact on biological control provision.

## Introduction

The expansion of chemical-intensive agriculture is regarded as the principal cause of widespread declines in beneficial biodiversity from agroecosystems, the degradation of ecosystem processes and economically important ecosystem services^1–3^. In an attempt to reverse trends in biodiversity loss, much research and conservation effort has focused on ways to restore semi-natural habitats back into agroecosystems and, in doing so, restore the valuable ecosystems services provided by agrobiodiversity^4–6^.

Positive relationships between biodiversity and ecosystem functioning are well documented in the literature where ecosystems with more diverse plant communities exhibit increased biodiversity at higher trophic levels^7,8^ and are functionally less susceptible to environmental stresses^9,10^. To this end, habitat management programs such as Agri-Environment Schemes (AES) have been implemented throughout Europe, aimed at protecting and enhancing the farmland environment^11^. A primary objective of these schemes has been to enhance the abundance and diversity of flowering plant species within arable systems^5^ via the creation of flower-rich habitats such as hedgerows, field-border plantings, cover crops and buffer strips^12^. This, in turn, will increase trophic system complexity and the diversity of species within the agricultural landscape that perform important roles i.e. the functional biodiversity, ultimately enhancing ecosystem service provision^13–15^. However, although such conservation measures have proved successful in boosting beneficial biodiversity^6,16^, the impact on ecological interactions and networks is often neglected^17,18^. Indeed, the sustainability of ecosystem service provisioning into the future depends on a clear understanding of how organisms provide ecosystem services, and how the different organisms interact with each other creating synergies or antagonisms^19–22^.

Relationships between ecosystem services may be antagonistic when the provisioning of one service increases at the expense of another service, or synergistic when the provisioning of two services increases simultaneously^19,22^. Competition between ecosystem service providers is one mechanism by which antagonistic relationships between ecosystem services may occur and has been the subject of increasing research attention^23–25^. When organisms share common resources, competitive interactions may result in reductions to the availability and quality of the resource to other foragers, detrimentally impacting the fitness of one or both competitors^26^. With agricultural landscapes largely homogenous and with low floral diversity^1^, competition over finite floral resources represents a strong selection pressure acting on flower-visiting species, many of which perform vital regulatory services including pollination and natural biological control. In the context of floral resources, an already exploited flower may possess diminished nectar and/or pollen resources. As an example, the flowers of buckwheat (*Fagopyrum esculentum*) may be depleted of nectar soon after midday as a result of flower-visiting insect species^25,27^. This makes the resource less rewarding for subsequent foragers; a form of competition referred to as exploitative competition which may, in turn, lead to competitive exclusion of the inferior competitor^18,28,29^. In addition, interference competition may occur whereby the superior competitor directly prevents the inferior competitor from accessing a shared resource^30,31^.

Competition between providers of the ecosystem service of pollination is well documented in the literature, with a primary focus on the interactions between domestic bees and wild bees, driven by concerns over declining wild bee populations^32–34^. Here, domestic bees (*Apis mellifera*) (Hymenoptera: Apidae) represent a superior competitor, outcompeting wild bee species by depleting and blocking access to shared floral resources. Despite a prior focus on bee species, competition with non-bee flower visitors is receiving increasing research attention. Hoverflies (Diptera: Syrphidae), for example, many species of which are predatory in their larval stage, are of particular interest due to their ability to perform pollination services as adults, but also biological control services as young^35^. However, recent research has revealed that adult hoverflies can be temporally displaced following competition with honeybees^24^. Much less is currently known about competitive interactions with parasitoid wasps; important biological control agents, many species of which rely on floral nectar as a source of sugar^36^. Recent research has shown that exploitative competition between heterospecifics over floral resources limits nutrient ingestion in a parasitoid wasp under laboratory conditions^25^. Here, parasitoid wasps (*Aphidius colemani*) (Hymenoptera: Braconidae) displayed reduced sugar content, notably glucose and fructose, when fed on flowers previously exploited by bumblebees (*Bombus terrestris*) (Hymenoptera: Apidae) and hoverflies (*Episyrphus balteatus*) (Diptera: Syrphidae) as opposed to unexploited flowers. Furthermore, without nectar intake, parasitoid longevity is significantly reduced to less than 24 h^37^. Ultimately, any resultant reduction in the fitness of a beneficial insect in response to diminished floral resources may, in turn, have consequences for the ecosystem services they provide.

With many temperate insects increasingly winter-active as a consequence of warming winters^38–41^, at a time when floral resources are scarce, novel competitive interactions between flower-visiting insects may occur in agroecosystems. While there are still few studies focusing on the winter period, plant diversification schemes including the use of flower strips may be implemented to provide targeted support for beneficial insects during the winter months and boost associated ecosystem service provision^42,43^. Damien et al.^42^ provided an initial study into the potential for flower strips to be implemented in winter months to support parasitoid wasps and ultimately enhance the ecosystem service of biological control. Here, flower strips were comprised of white mustard (*Sinapis alba*); a species capable of producing flowers and thus providing a nectar source during temperate autumn and winter months^42^. The study revealed the potential of winter flower strips to significantly increase parasitism rate, supporting the ‘parasitoid nectar provision hypothesis’^44^ which posits that increased plant diversification increases nectar availability for parasitoids, leading to a reduction in pest pressure. However, whilst initial research suggests the possible benefits of winter flower strips, very little is currently known about the potential for competitive interactions between winter-active parasitoids and pollinators.

The current study employed winter field trials and laboratory cage experiments to investigate the impact of winter flower strips on the biological control of aphids performed by parasitoid wasps, and the potential for competitive interactions between winter-active parasitoid wasps and pollinators and resultant implications for this ecosystem service. It was hypothesized that (1) winter flowering strips will increase parasitoid abundance and aphid parasitism in the field in line with the parasitoid nectar provision hypothesis; (2) winter pollinator abundance will be too low to compete with parasitoids and thus parasitism rate will not be affected by pollinator presence; (3) In laboratory cage experiments where pollinator abundance can be artificially manipulated, there will be a negative correlation between pollinator number and the parasitism rate of cereal aphids.

## Results

### Winter field trials

**Figure 1:**
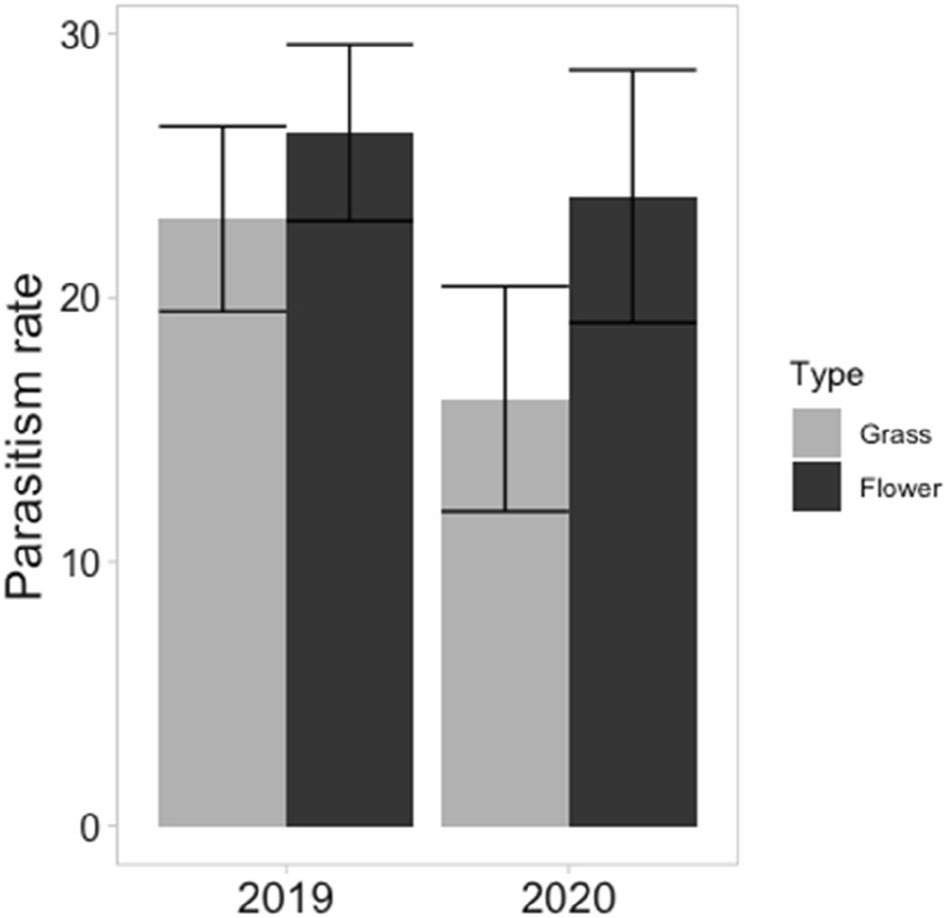
Mean percentage parasitism (± standard error) of cereal aphids in the field depending on the field edge in the winter field trial of 2019/20 and 2020/21. Grey bars represent the parasitism rate of aphids collected near the field edge adjacent to a grassy margin of spontaneous herbaceous plants composed of primarily Poaceae. Black bars represent the parasitism rate of aphids collected near the field edge adjacent to the flower strip comprised of white mustard (*Sinapis alba*), fodder radish (*Raphanus sativus*), buckwheat (*Fagopyrum esculentum*) and a Fabaceae species *(Vicia faba* or *V. sativa*).

A total of 2410 aphids were collected from the field across the two sampling years, resulting in the formation of 501 aphid mummies. Of the adult parasitoids emerging from the aphid mummies, 261 were identified to species level. These comprised 15 *Aphidius avenae* (5.7%), 59 *A. ervi* (22.6%), 39 *A. matricariae* (14.9%), 148 *A. rhopalosiphi* (56.7%)*.* There was no significant difference in the parasitism rate of cereal aphids in field edges adjacent to flower strips and field edges adjacent to grassy margins (Fig. 1) (*X*^2^ = 2.042, df = 1, *p* = 0.153). Furthermore, there was no effect of year on parasitism rate (*X*^2^ = 3.056, df = 1, *p* = 0.080), nor a significant interaction between field edge and year (*X*^2^ = 0.308, df = 1, *p* = 0.578). Across the two years, a total of 268 transect walks were conducted; 144 in the flower strips and 124 in the grassy margins of the 22 study fields. A total of 180 pollinators were observed during 63 of the 144 transect walks conducted in the flower strips which included 13 individuals of the *Apis mellifera*, 40 individuals of the genus *Bombus*, 1 small wild bee (< 1 cm in size), 16 large wild bees (> 1 cm in size), 35 individuals of aphidophagous hoverfly species, and 75 individuals of non-aphidophagous hoverfly species. Of the 124 transect walks conducted in the grassy margins, only 1 pollinator of the genus *Bombus* was observed. For this reason, this one observation obtained in the grassy margin was excluded from the subsequent analysis on parasitism rate. Overall, there was no significant effect of pollinator abundance on the parasitism rate of cereal aphids in the field next to the flower strip (*X*^2^ = 0.319, df = 1, *p* = 0.572).

### Laboratory cage experiments

**Figure 2:**
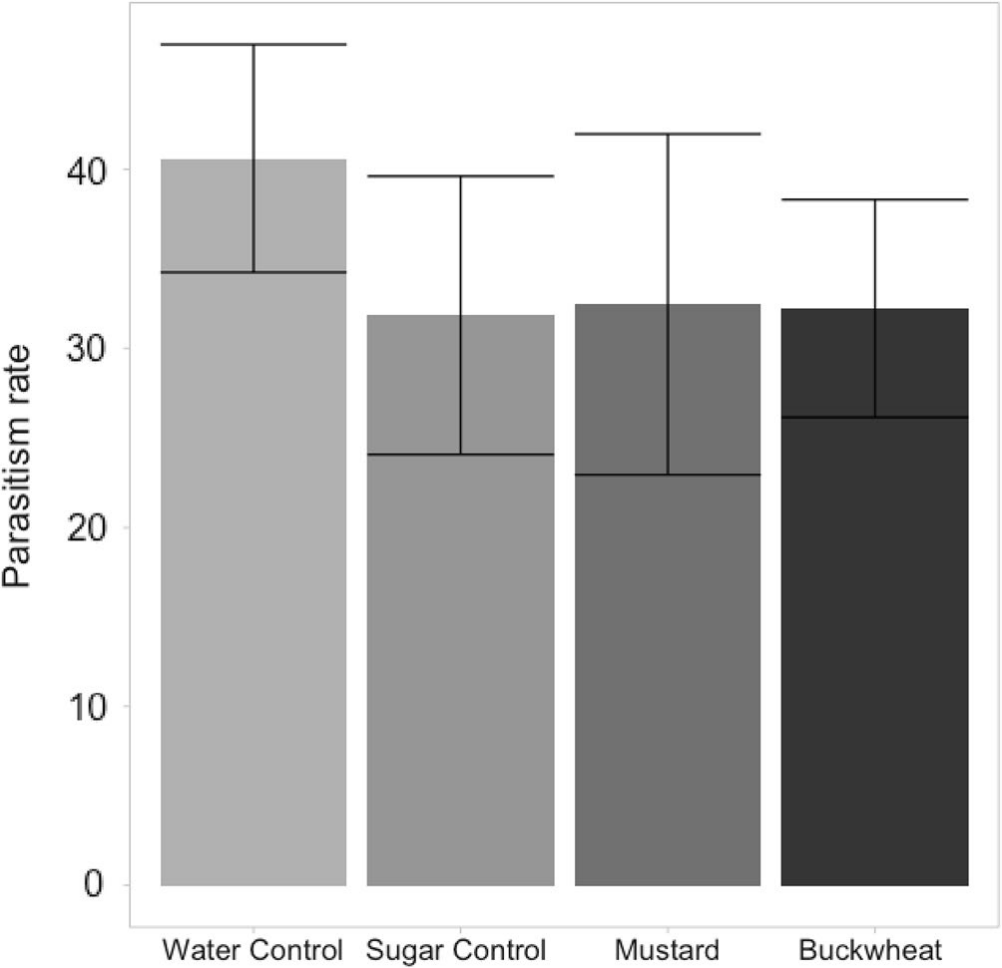
Mean percentage parasitism of *Sitobion avenae* (± standard error) by *Aphidius ervi* in the presence of either distilled water (negative control), a 1 M sucrose solution (positive control), white mustard (*Sinapis alba*), or buckwheat (*Fagopyrum esculentum*).

**Figure 3:**
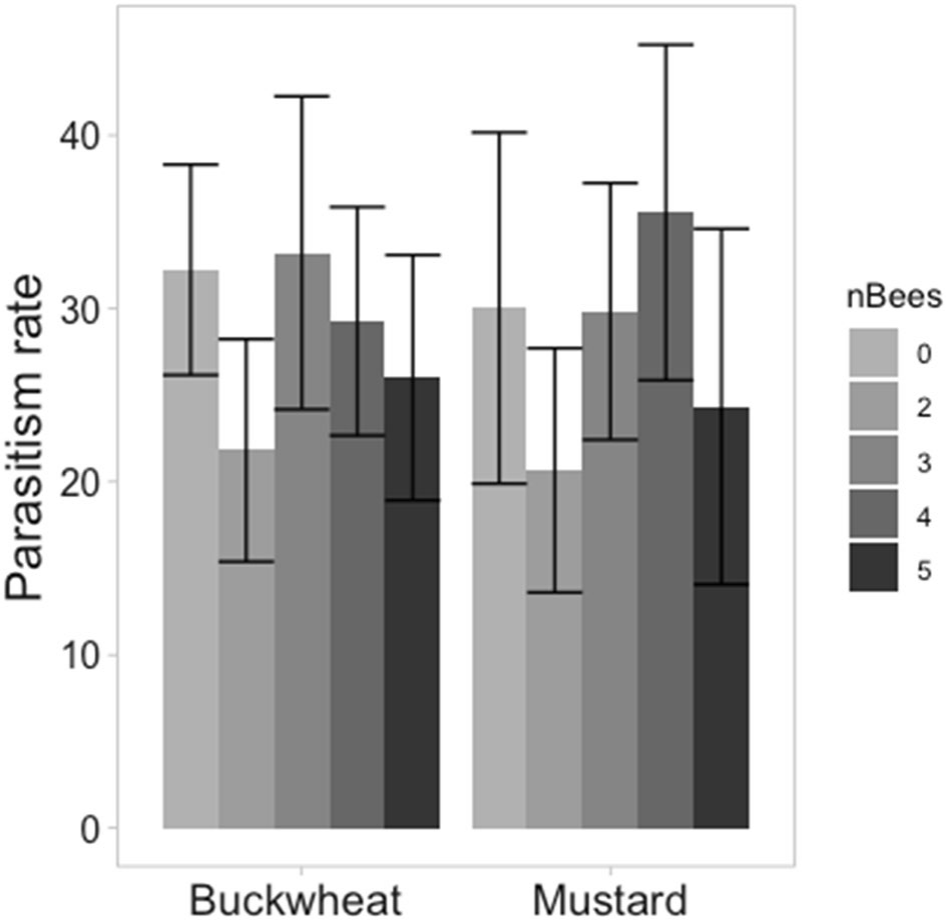
Mean percentage parasitism of *Sitobion avenae* (± standard error) by *Aphidius ervi* in the presence of white mustard (*Sinapis alba*) or buckwheat (*Fagopyrum esculentum*), and either 0, 2, 3, 4, or 5 worker *Bombus terrestris*.

There was no significant effect of floral resource on the parasitism rate of *Sitobion avenae* (Hemiptera: Aphididae) by *Aphidius ervi* (Hymenoptera: Braconidae) in the absence of *B. terrestris* (*X*^2^ = 0.835, df = 1, *p* = 0.841) (Fig. 2). Bee (*B. terrestris*) density had no significant effect on the parasitism rate of *S. avenae* by *A. ervi* (*X*^2^ = 0.343, df = 1, *p* = 0.557) (Fig. 3). Furthermore, there was no significant effect of floral resource on parasitism rate in the presence of *B. terrestris* (*X*^2^ = 1.728, df = 1, *p* = 0.189), nor a significant interaction between floral resource and bee number (*X*^2^ = 1.499, df = 1, *p* = 0.221).

## Discussion

The current study employed winter field trials and laboratory cage experiments to investigate the impact of winter flower strips on the ecosystem service of biological control performed by parasitoid wasps and the potential for competitive interactions between winter-active parasitoid wasps and pollinating insects. It was hypothesized that winter flowering strips would have a positive effect on parasitoid wasps in the field, in line with the parasitoid nectar provision hypothesis^44^, resulting in enhanced parasitism rates of cereal aphids (hypothesis 1). However, contrary to our first hypothesis, parasitism rates of cereal aphids in the field were not enhanced near the flower strips, thus disproving hypothesis 1. Due to the lack of positive effect of winter flower strips on aphid parasitism rate, one possible explanation is that competition may have occurred between parasitoids and winter-active pollinators over finite floral resources. However, we hypothesized that pollinator abundance in the field would presently be too low to compete with parasitoids and thus parasitism rate would not be affected by pollinator presence. The results of the winter field trials revealed that, whilst pollinators were present during winter months, pollinator abundance in the field was low and did not significantly impact the parasitism rate of cereal aphids, thus supporting hypothesis 2. Finally, in laboratory cage experiments, where the abundance of pollinators could be artificially manipulated, we hypothesized that there would be a negative correlation between pollinator abundance and the parasitism rate of cereal aphids. However, this was not supported and the current study found no effect of pollinator abundance on the parasitism rate of aphids, thus disproving hypothesis 3. The current study therefore suggests that exploitative competition is not occurring between winter-active pollinators and parasitoid wasps, with no detrimental impact on the ecosystem service of aphid biological control performed by parasitoid wasps. Thus, the presence of winter-active pollinators is not responsible for the lack of a positive impact of the winter flower strip on parasitism rate.

In the current study, the presence of a winter flower strip comprising white mustard (*S. alba*), fodder radish (*R. sativus*), buckwheat (*F. esculentum*) and a Fabaceae species (*V. faba* or *V. sativa*) did not significantly impact the parasitism rate of cereal aphids in the adjacent cereal field when compared to the field edge bordered by a grassy margin. It was initially considered that, as insects become more active during winter months^38,40,41,43^, increases in pollinator abundance in the field could lead to competition over floral resources, thus offering a potential explanation for the lack of positive impact the winter flower strip had on aphid parasitism rate. However, this was soon refuted in the current study since pollinator abundance had no significant effect on aphid parasitism rate. Thus, pollinator abundance is not responsible for the absence of a positive impact of the winter flower strip on parasitism rate in the current study. However, our lack of support for the parasitoid nectar provision hypothesis is not entirely unexpected and, the published literature, as recently reviewed by Heimpel^45^, suggests that nectar provisioning commonly fails to improve biological control. Possible reasons for these failures in the literature include a lack of sugar limitation experienced by the parasitoid or that the supplementary nectar benefits the pest more than it does the parasitoid^45^. A novel additional hypothesis put forward by Heimpel^45^ suggests that sugar feeding may counterintuitively encourage parasitoid dispersal rather than retention in vicinity of the flower strip. Whilst this is still to be thoroughly tested in the field, a study by Wäckers^46^ involving wind tunnels found that sugar-fed parasitoids (*Cotesia rubecula*) (Hymenoptera: Braconidae) engaged in flight more than unfed parasitoids, offering support to Heimpel’s^45^ hypothesis.

Interestingly our study, which found no positive effect of the winter flower strip on parasitism rate, contradicts a previous study by Damien et al.^42^ conducted in the same geographic region. Here, authors found that winter flower strips comprised of white mustard (*S. alba*) led to a 13% increase in parasitism rate of aphids in bordering cereal fields. One possible explanation for the discrepancy between the current study and that of Damien et al.^42^ could be that the flower mix used in the present study (*S. alba*, *R. sativus*, *F. esculatum*, and a Fabaceae species) was not as attractive to parasitoids as the flower strips comprised of solely *S. alba* used by Damien et al.^42^ Parasitoids of a number of genera have been shown to be preferentially attracted to the colour yellow^46–48^. The addition of *R. sativus*, *F. esculatum*, and a Fabaceae species to the flower mix may have thus diluted the stimulus of the yellow flowers of *S. alba*, reducing the overall attractiveness of the flower strip. Such variation in the attractiveness of flower strips to parasitoids may also provide explanation for the varied success of flower strips in promoting biological control reported in the literature^45^.

During the winter field trials of 2019/20 and 2020/21, a total of 180 pollinators were observed during transect surveys with a dominance of non-aphidophagous hoverfly species, predominantly *Eristalis* species, and bees of the genus *Bombus*. This observation is in line with what is known about the thermal constraints of pollinators which limits their foraging behaviour during winter months. *Bombus* species, for example, are well adapted to foraging at lower temperatures in relation to other bee species such as the honeybee (*A. mellifera*), making them important pollinators early in the year^49,50^. *B. terrestris* workers, for example, have been shown to survive brief exposures to temperatures close to their supercooling point (the temperature at which spontaneous freezing of the insect occurs), with over 80% of an experimental population surviving a 2 h exposure to − 5 °C. Furthermore, the LTime_50_ (the time taken to kill 50% of the population) for *B. terrestris* queens was found to be over 25 days at 0 °C^51^. Hoverflies are also reported to be more active in colder climates^52^, leading authors to conclude that hoverflies would be more active at colder temperatures than their bee counterparts. This is confirmed in the current study with the dominance of hoverflies and bees of the genus *Bombus* in the winter field trials. However, although pollinators were present in the winter flower strips, the abundance of pollinators had no effect on the parasitism rate of cereal aphids by *Aphidius* parasitoids in the field. It is possible that the abundance of winter-active pollinators in the field is presently too low to result in exploitative competition between pollinators and parasitoid wasps.

To further test this, we conducted laboratory cage experiments where the density of bees (*B. terrestris*) could be artificially increased beyond what is experienced in the field. However, even at densities as high as 5 bees per 45 cm^3^ cage^53^, bee abundance had no significant effect on the parasitism rate of aphids by the parasitoid wasp. This result was irrespective of whether the floral resource present was buckwheat or white mustard. This finding, in conjunction with the results of the winter field trials, suggests that exploitative competition between pollinators and parasitoid wasps is unlikely, even in the event of increased winter-activity and insect abundance. It is, however, possible that parasitoids are not feeding on the nectar, perhaps as a consequence of a reduced metabolic rate at low temperatures^54^, resulting in parasitoid activity being less limited by food availability under winter conditions. The results of our cage experiments in the absence of bees offers support to this hypothesis where we observed no significant difference in parasitism rate by parasitoids in the presence of a sugar source (white mustard, buckwheat or a 1 M sucrose solution) and in the absence of a sugar source (distilled water only). These results provide support to the possibility that parasitoids are not utilising floral resources and thus parasitoid activity under winter conditions is not limited by food availability.

Although evidence for exploitative competition was lacking in the current study, we cannot rule out the occurrence of other forms of competition such as interference competition arising from territoriality or chemical competition, leading to displacement of the inferior competitor^30,31^. Indeed, ecological mechanisms such as resource partitioning may come into play to limit competition and enable the species to coexist and share resources^55^. In a recent study into floral resource exploitation by different pollinator morphogroups, spatio-temporal variation in resource use was observed^24^. In the study of Jeavons et al. the honeybee (*A. mellifera*) was found to be the dominant flower visitor^24^. As a consequence of honeybee presence, hoverflies foraged earlier in the day, whilst wild bee species preferentially foraged on extrafloral nectaries. Such alterations to resource use are plastic and may occur over short time frames in response to the presence of a superior competitor. Any spatial^56^ or temporal^57^ displacement of the inferior competitor will act to minimize competition with the dominant competitor, enabling the inferior competitor to be locally maintained. It is therefore possible that spatio-temporal variation in resource use between pollinators such as those of the genus *Bombus* and parasitoid wasps of the genus *Aphidius* is occurring, either temporally displacing parasitoids to forage at less-preferred times of the day, or spatially displacing parasitoids to feed on the less profitable sugar source, aphid honeydew^58^. However, whilst it is known that honeydew may represent a significant proportion of the diet of *Aphidius* wasps under spring and summer conditions^59^, the importance of aphid honeydew to *Aphidius* wasps under winter conditions is currently unknown. The use of this less profitable sugar source in the presence of the flower strip could represent an optimal choice should the cost of dispersal out of the cereal field outweigh the benefit of the richer nectar source to be obtained in the adjacent flower strip, particularly at low temperatures where reduced metabolic rate may reduce activity^54^. Nonetheless, any spatio-temporal displacement of parasitoid wasps would enable both groups of beneficial insects to co-exist and would explain the lack of effect bee abundance had on the ecosystem service of aphid biological control in the current study. Further studies are required to determine if spatial or temporal displacement of parasitoid wasps is occurring in response to winter-active pollinators.

Ultimately, this study suggests that whilst floral resources may be implemented during winter to support nectar-feeding insects at a time when floral resources are scarce, the impact of plant diversification measures on ecological interactions and the wider network cannot be overlooked. Any novel competitive interactions generated over the floral resources could act to undermine the delivery of ecosystem services by winter-active insects. However, at least for winter-active pollinators primarily of the genus *Bombus* and *Aphidius* parasitoid wasps, exploitative competition is not occurring, with no detrimental impact on the ecosystem service of aphid biological control performed by parasitoid wasps.

## Materials and methods

### Winter field trials

#### Study site

Winter field trials were conducted in the Brittany region of North Western France during two growing seasons (2019/20 and 2020/21). A total of 10 fields were selected for the 2019/20 winter field season and 12 fields for the 2020/21 winter field season. Fields were separated by at least 850 m and all fields had been previously sown with either wheat or barley in late October to mid-November. A paired experimental design was employed to compare the area of a cereal field directly adjacent to a flower strip to the area adjacent to a grassy margin, separated by at least 50 m^42^. For this, a flower strip comprising the winter-flowering species of white mustard (*Sinapis alba*, var. Rota and Signal, 26% by weight), fodder radish (*Raphanus sativus*, var. Litinia, 27% by weight), buckwheat (*Fagopyrum esculatum*, var. Billy, 40% by weight), and a Fabaceae species (*Vicia sativa*, var. Gravesa, or *Vicia faba*, var. Bobas and Fernando 7% by weight) was sown adjacent to each cereal field in mid-August to early September. These selected species have late and long-lasting flowering periods and produce nectar or extra-floral nectar at winter temperatures^40^. Each flower strip had a minimum surface area of 0.5 hectares (W = 30 m; L = 160 m minimum), further acting as a cover crop to protect bare soil from erosion during the winter months. On the opposite side of the field to the flower strip was a grassy margin of spontaneous herbaceous plants composed of primarily Poaceae at least one meter in width. Fields were visited once every 3 weeks from 28th November 2019 to 5th March 2020 for the 2019/20 sampling period, and 23rd November 2020 to 12th March 2021 for the 2020/21 sampling period. This resulted in 6 distinct sampling sessions per year. During each field visit, parasitoids and pollinators were sampled as detailed below.

#### Parasitoid sampling

Aphids were collected by hand in the field within 10 m from the field edge adjacent to either the flower strip or the grassy margin. Individual cereal plants were inspected by eye until an aphid was found. On finding an aphid, the cereal leaf on which the aphid was feeding was cut and placed in a 50 ml Falcon tube, along with any additional aphids feeding on the same leaf. A total of 10 leaves containing at least one feeding aphid were collected per sampling area. In the event that 10 leaves containing aphids could not be found, the search was terminated at 30 min. All collected aphids were returned to the laboratory.

On returning to the laboratory, collected aphids were counted and identified to species level (*S. avenae*, *Metopolophium dirhodum*, *Rhopalosiphum padi*). Once sorted, aphids were placed in microcages (L = 16 cm, Ø = 4 cm) containing wheat plantlets (*Triticum aestivum*) grown in vermiculite for a period of 5 days prior to their use. Microcages were then transferred to a temperature controlled room set to 20 °C ± 1 °C and LD 16: 8 h and checked daily for the development of aphid mummies (a parasitized aphid containing the parasitoid pupa). The number of aphid mummies formed was subsequently used to estimate parasitism rate using the formula below:

$$\frac{{{\text{total}}\,{\text{ number }}\,{\text{of }}\,{\text{mummies }}\,{\text{formed}}}}{{{\text{total }}\,{\text{number }}\,{\text{of}}\,{\text{ aphids}} + {\text{mummies }}\,{\text{collected }}\,{\text{in }}\,{\text{the}}\,{\text{ field}}}}$$

All resultant aphid mummies were isolated individually in gelatine capsules and maintained in the laboratory at 20 ± 1 °C until parasitoid emergence. On emergence, all adult parasitoids were preserved in 70% ethanol and identified to species level^60^.

#### Pollinator sampling

Two 50 m transects were established in each field: one in the flower strip and one in the grassy margin. All transects were established 5 m from the border between the crop and the flower strip or the crop and the grassy margin. A transect walk was conducted along each transect for a duration of 20 min and all flower-visiting insects were scored as one of the following morphogroups: bumble bees (*Bombus* spp), honey bees (*Apis mellifera*), large wild bees (> 1 cm), small wild bees (< 1 cm), adult non-aphidophagous hoverflies, and adult aphidophagous hoverflies^24^. Flower-visiting insects were scored as present if observed foraging on the flower, foraging on extra-floral nectar, resting on the plant, or flying between floral resources. Temperature in the shade was recorded using a digital thermometer and was used to inform the conditions of subsequent laboratory cage experiments detailed below.

### Laboratory cage experiments

Laboratory cage experiments were conducted to investigate potential competition between winter-active parasitoid wasps and pollinators over floral resources, with the experimental set-up enabling fine control over pollinator density and thus manipulation of competition pressure between the two groups of insects. Of the species of pollinators and parasitoids sampled during the winter field trials, *B. terrestris* was chosen as the study pollinator and *A. ervi* as the study parasitoid species for use in cage experiments due to the ease with which these species can be obtained from horticultural companies. *A. ervi* was reared on *S. avenae* as the host aphid. All insect species were obtained from Royal Brinkman, UK, and maintained in the laboratory at 15 °C ± 1 °C and LD 12: 12 h to represent winter conditions. The temperature of 15 °C was chosen based on the average temperature recorded in the shade during the pollinator transects conducted in 2019/20 and 2020/21 when pollinators were observed in flight. The temperature of 15 °C thus represents an ecologically relevant winter temperature for the study region of Brittany, France, when pollinators would be active. White mustard (*S. alba*) and buckwheat (*F. esculentum*) were selected as the floral resources. White mustard and buckwheat differ in their nectar composition and sucrose:hexose ratio, with white mustard having a hexose dominant nectar and buckwheat a sucrose dominant nectar^61^. Furthermore, the two species differ in their attractiveness to parasitoids, and in their effect on parasitoid fitness^62,63^.

#### Insect rearing

Colonies of *S. avenae*, *A. ervi*, and *B. terrestris* were maintained in the laboratory under conditions of 15 °C ± 1 °C and LD 12: 12 h within separate BugDorm fine mesh tents (60 cm × 60 cm × 60 cm). *S. avenae* were reared on winter wheat (*T. aestivum*) grown in vermiculite. *A. ervi* were maintained on *S. avenae* and fed on a solution of honey and water. Pots of winter wheat infested with *S. avenae* were added to the cages containing the parasitoid wasps on a weekly basis to provide the wasps with a continual supply of hosts. *B. terrestris w*ere fed on a 1 M sucrose solution, a supply of fresh water and shop-bought bee pollen (Sevenhills Wholefoods, UK) provided twice per week. A 1 M sucrose concentration is considered to be representative of the sugar concentration found in floral nectar^63–65^. To select bees for use in experiments, worker bees actively foraging outside the hive were selected on the day of the experiment and transferred individually to the experimental cage within a plastic tube (Ø = 4 cm, L = 7 cm). To obtain parasitoids for use in experiments, aphid mummies were isolated in gelatine capsules until emergence to ensure that all emerging parasitoids were unfed and unmated since unmated females are more willing to feed than mated females^62^. The capsules were checked twice daily for emergence. Following emergence, only female parasitoids were retained and used in experiments on the day of emergence to ensure that all parasitoids were < 24 h old at the start of the experiment.

#### Plant growth

White mustard (*S. alba*) and buckwheat (*F. esculentum*) were grown within University glasshouses. All plants used in experiments were harvested at 7 to 9 weeks old to represent the age at which the produced nectar is of the highest quality and quantity^66,67^. To standardise the amount of available nectar, a total of 20 white mustard and 60 buckwheat inflorescences were selected for use in each cage experiment to account for variation in nectar production^66,67^. Fresh inflorescences were cut from plants and placed in Oasis^®^ floral foam saturated with water enabling continued nectar production for the duration of the experiment^63^.

#### Cage experiment investigating the effect of floral resource on parasitism rate

Cage experiments were performed within BugDorm fine mesh cages (45 × 45 × 45 cm) under laboratory conditions of 15 °C ± 1 °C and LD 12: 12 h. All cages were set up to contain a sugar source of either 20 inflorescences of white mustard (white mustard treatment), 60 inflorescences of buckwheat (buckwheat treatment), cotton wool soaked in a 1 M sucrose solution (positive control), or cotton wool soaked in distilled water (negative control). A pot of wheat infested with 50 L2 and L3 larval stage aphids, representing the preferred age host^68^, was placed in the centre of each cage. Two virgin female parasitoid wasps (< 24 h old) were then released into the cage and the experiment was started. The cage experiment was run for a period of 24 h. All cage experiments commenced at approximately 2:30 pm and thus concluded at 2:30 pm the following day. Following the 24 h experimental period, the pot of wheat was removed from each cage and sealed within a perforated plastic bag to enable the wheat to continue to grow and the aphids to continue to feed. Wheat pots were isolated in this way for a period of 2–3 weeks until aphid mummy formation. Following aphid mummy formation, the number of mummies formed per 50 aphids was counted and parasitism rate calculated. Cage experiments were repeated to create 10 replicates of each treatment group.

#### Cage experiment investigating the effect of bee density on parasitism rate

Cage experiments were performed using the experimental set up detailed above, with the addition of *B. terrestris*. Worker bees were released into the cages to provide abundances of either 0, 2, 3, 4, or 5 bees per cage. Concurrently, 2 virgin female parasitoid wasps (< 24 h old) were released into the cage and the experiment was started. The cage experiment was run for a period of 24 h. All cage experiments commenced at approximately 2:30 pm and thus concluded at 2:30 pm the following day. Following the 24 h experimental period, worker bees were removed from the cage and the pollen baskets checked by eye for the presence of pollen as an indication that foraging activity had occurred. The pot of wheat was removed from each cage and sealed within a perforated plastic bag. Wheat pots were isolated until aphid mummy formation. Following aphid mummy formation, the number of mummies formed per 50 aphids was counted and parasitism rate calculated as explained previously. Cage experiments were repeated to create 10 replicates of each treatment group.

### Statistical analyses

Parasitism rates in winter cereal fields were analysed using Generalized linear mixed models (GLMMs) with a binomial distribution and a logit link whereby “strip type” and “sampling sessions” were considered as fixed factors as well as their interaction. “field” was considered as a random factor to account for the dependency of observation. GLMMs were built with the functions glmer of the R package lme4^69^. To investigate the effect of pollinator abundance on aphid parasitism rate in the field next to the flower strip, the abundance of pollinators at an instant *t* and the parasitism rate an instant *t* + 3–4 weeks was implemented to account for the time lag in aphid mummy formation from the point of parasitism^70^. Pollinator abundance was linked to parasitism rate *t* + 3–4 weeks for 9 sampling sessions across the two years, resulting in 89 data points from discrete transects walks performed in the study fields. Parasitism rate data were once again analysed using Generalized linear mixed models (GLMMs) with a binomial distribution and a logit link and abundance of pollinators as an explanatory variable, whereby “field” was considered as a random factor. Parasitism rates in the cage experiments were analysed using GLMs also with a binomial distribution and a logit link. One model was built with “food resource” (flowers: buckwheat or mustard, positive control and negative control) as a fixed factor. A second model was built to analyze parasitism rate according to bee competition (0, 2, 3, etc.) and flower species (mustard or buckwheat) and their interaction. The significance of fixed effects in the models fitted with the maximum likelihood was assessed by comparing a model with and without the fixed effect using likelihood-ratio tests (LRT) against a χ2 distribution^71^. All models were validated by analysing their residuals with the DHARMa package^72^. All statistical analyses were performed with R Version 4.1.2.

## Data Availability

The datasets used and analysed during the current study are available from the corresponding author on reasonable request.
